# The CONUT score is associated with the pathologic grade in non-small cell lung cancer

**DOI:** 10.1007/s00595-024-02860-8

**Published:** 2024-05-06

**Authors:** Ken Onodera, Hirotsugu Notsuda, Tatsuaki Watanabe, Yui Watanabe, Takaya Suzuki, Takashi Hirama, Hisashi Oishi, Hiromichi Niikawa, Masafumi Noda, Yoshinori Okada

**Affiliations:** Department of Thoracic Surgery, Institute of Development, Aging and Cancer, Tohoku University, Seiryomachi 4-1, Aoba-ku, Sendai, Miyagi 980-8575 Japan

**Keywords:** Lung cancer, NSCLC, Early stage, Nutritional status, CONUT

## Abstract

**Purpose:**

Nutritional scores have been reported to be useful prognostic factors for various cancers. This study evaluated the usefulness of the preoperative controlling nutritional status (CONUT) score as a predictor of recurrence of non-small cell lung cancer (NSCLC).

**Methods:**

The present study included 422 patients with stage I–IIIA NSCLC who underwent complete resection at Tohoku University Hospital between January 2010 and December 2016. The patients were divided into the low-CONUT and high-CONUT groups based on their CONUT scores. Overall survival (OS), recurrence-free survival (RFS), and cumulative recurrence rates in the low- and high-CONUT groups were evaluated retrospectively.

**Results:**

One hundred forty-seven patients (34.8%) were assigned to the high-CONUT group. The high-CONUT group had a significantly worse performance status, pleural invasion, vascular invasion, and lung metastasis. In the whole cohort, the low-CONUT group showed better overall survival, recurrence-free survival, and a low cumulative recurrence rate in comparison to the high-CONUT group. There was no significant difference in prognosis or recurrence between the low- and high-CONUT groups after propensity score matching.

**Conclusion:**

Patients with a high CONUT score may be at high risk of recurrence because of the high frequency of pleural invasion, vascular invasion, and lung metastasis.

## Introduction

Non-small cell lung cancer (NSCLC) is the leading cause of cancer-related death worldwide. The 5-year overall survival (OS) rates for patients with pathological stage IA1/IA2/IA3/IB early-stage lung cancer are 90%, 85%, 80%, and 73%, respectively. There is still room for improvement [1]. Lung cancer staging, which indicates disease progression and malignancy, is determined using the TNM classification. However, even among patients with the same stage of the disease, there is wide variation in the rate of recurrence after resection. Therefore, the current TNM staging system, based on pathological findings, may be inadequate for predicting the risk of recurrence.

In addition to the factors included in the TNM classification, several other risk factors for recurrence have been reported. Pathological findings such as histological differentiation, vascular invasion, lymphatic invasion, and pleural invasion have been reported as poor prognostic factors for disease-free survival (DFS) [2–4]. As for clinical factors, tumor markers such as carcinoembryonic antigen and the standardized uptake value of positron emission tomography (PET) of the primary lesions, have also been reported to be risk factors for recurrence [5, 6]. To improve the prognosis of early-stage NSCLC, it is important to identify other risk factors for recurrence.

In recent years, the efficacy of several immune checkpoint inhibitors (ICIs) against advanced lung cancer has been confirmed, including nivolumab (Ono Pharmaceutical, Osaka, Japan), a PD-1 inhibitor [7]. This trend has also influenced perioperative therapy for NSCLC. The results of some trials, such as the Impower010 and Checkmate816 trials, have been reported [8, 9]. All showed favorable results. ICIs restore the host’s immune functions against cancer cells by impeding immune escape mechanisms. In basic research, the importance of regulatory T cells in suppressing anticancer immunity was already demonstrated in the late 1990s [10, 11]. Therefore, the patient’s general condition, including their immune status, may be closely related to cancer progression and recurrence.

The controlling nutritional status (CONUT), which consists of the sum of the scores for albumin, total cholesterol, and lymphocyte count, has been reported to be an indicator of the immune-nutritional status [12]. The usefulness of the CONUT score as a prognostic factor in NSCLC has been reported [13–15], but its effectiveness is primarily observed in DFS, OS, and cancer-specific survival. Undernourished patients without cancer may have a poor prognosis, and the relevance of the CONUT score to recurrence is unclear. This study aimed to clarify the impact of the CONUT score on the risk of recurrence in patients with completely resected NSCLC.

## Methods

### Ethical statement

The institutional review board of Tohoku University Hospital in Sendai, Japan, approved this retrospective review of a prospective database (approval date: September 28, 2020; approval code: 2021-1-912-1) and waived the requirement for informed consent.

### Patients

**Fig. 1:**
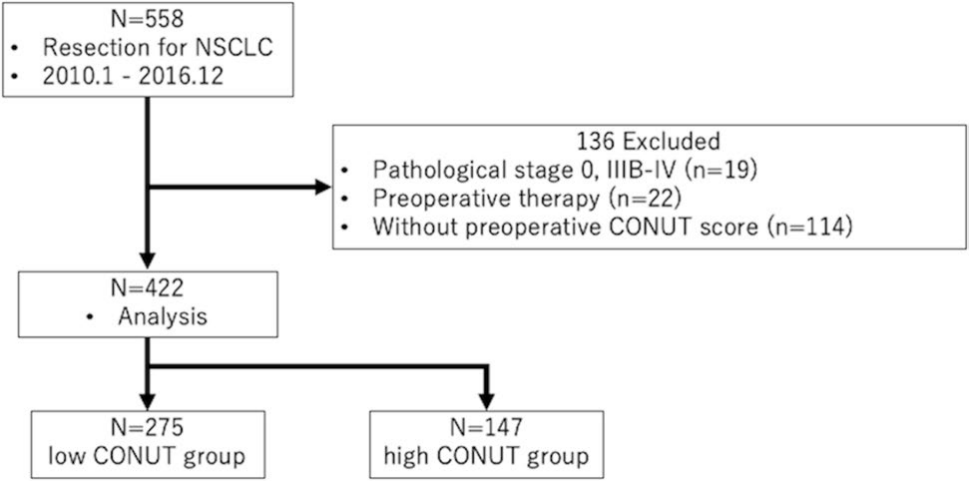
Consort diagram. *CONUT* controlling nutritional status

Between January 2010 and December 2016, 564 patients underwent lung resection for NSCLC at Tohoku University Hospital. Patients with pathological stages I–IIIA were included. Patients who received preoperative treatment or had a deficient preoperative CONUT score were excluded. This resulted in a total of 422 patients (Fig. 1). The selection of surgical procedure and the administration of platinum-based adjuvant chemotherapy were determined by the treating physician based on the guidelines for lung cancer treatment. Patients were regularly evaluated at three-month intervals for the first 2 years, and typically at 6-month intervals thereafter. Follow-up evaluations included a physical examination, chest radiography, blood examination (including tumor markers), and chest computed tomography (CT). Further evaluations, including brain magnetic resonance imaging (MRI) and PET/CT, were performed whenever symptoms or signs of recurrence were detected. All patients were followed-up for at least 5 years after surgery. Recurrence was diagnosed based on compatible physical examination and diagnostic imaging findings, and histological confirmation was obtained when clinically indicated.

### Clinicopathologic evaluations

All patient information was extracted from electronic medical records and databases, and clinical and pathological staging was reassessed according to the eighth edition of the TNM classification [1]. Histological typing was performed according to the World Health Organization classification [16]. The preoperative nutritional status was scored and evaluated using the CONUT score, which represents the sum of the scores for albumin, total cholesterol, and lymphocyte count [12] based on preoperative blood tests. The CONUT score is an index that evaluates the nutritional status. It is calculated as the sum of scores for albumin (0 to 6), total cholesterol (0 to 3), and lymphocyte count (0 to 3). CONUT scores of 0–1, 2–4, 5–8, and 9–12 are classified as normal, mild, moderate, and severe, respectively. In the present study, we classified patients with CONUT scores of 0–1 (normal nutrition) into the low-CONUT group, and those with scores of ≥ 2 (low nutrition) into the high-CONUT group based on previous reports**.**

### Statistical analysis

OS was defined as the period from surgery to death from any cause or the date of the last follow-up examination. Recurrence-free survival (RFS) was defined as the period from surgery to recurrence, death from any cause, or censorship at the final follow-up examination. The cumulative recurrence rate (CRR) was defined as the period from surgery to recurrence or censored at the last follow-up examination. Patient characteristics were summarized using frequencies and descriptive statistics, such as medians and ranges. OS, RFS, and CRR were estimated using the Kaplan–Meier method. Hazard ratios (HRs) and confidence intervals (CIs) were estimated using univariate and multivariate proportional hazard models.

**Fig. 2:**
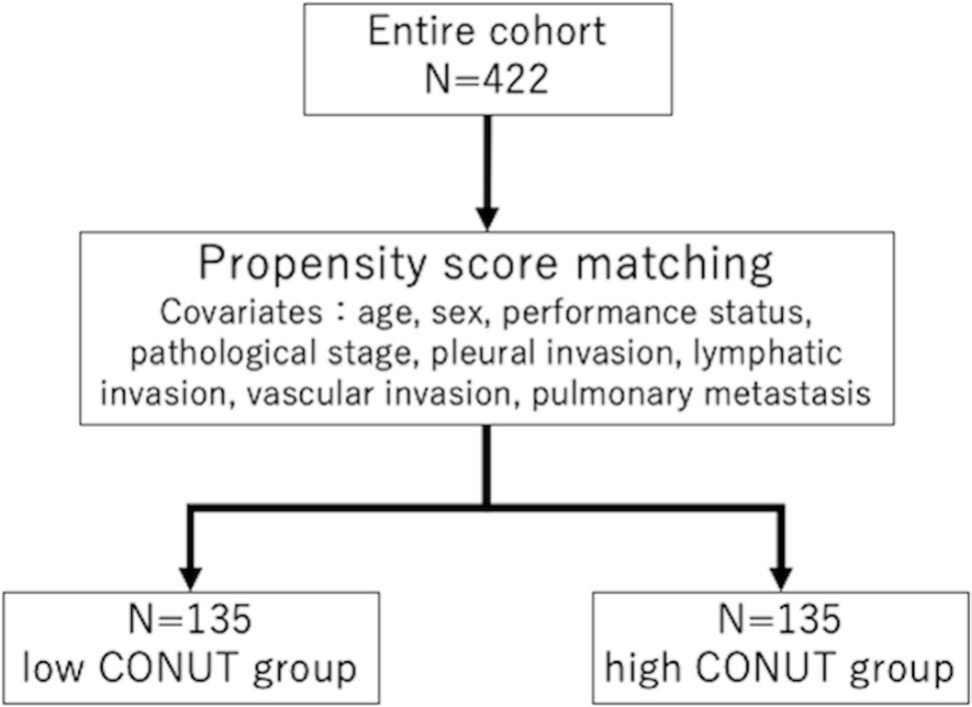
Propensity score matching. *CONUT* controlling nutritional status

To eliminate as much bias as possible, propensity score matching (PSM) was performed, with age, sex, performance status (PS), pathological stage, pleural invasion, lymphatic invasion, vascular invasion, and pulmonary metastasis included as covariates. A total of 270 patients were included in the propensity score-matched cohort (Fig. 2).

All statistical analyses were performed using JMP Pro version 17.1.0 (SAS Institute, Cary, NC, USA). Statistical significance was set at *p* < 0.05, without adjustment for multiple tests.

## Results

### Patient characteristics

The clinicopathological characteristics of the patients (male, *n* = 236; female, *n* = 186; median age, 69 years; range 22–96) are shown in Tables 1 and 2. There were 45 patients with PS ≥ 1, 278 were ever-smokers, 350 underwent lobectomy, 313 had adenocarcinoma, 107 had other histologic types, and 309, 73, and 40 had pathological stages I, II, and III, respectively. One hundred four patients had pleural invasion, 156 had vascular invasion, 114 had lymphatic invasion, and 13 had pulmonary metastasis. Recurrence was observed in 102 patients. The high-CONUT group had a significantly poorer PS and increased rates of pleural invasion, vascular invasion, and lung metastasis.

**Table 1:** Patient characteristics

Characteristic	Entire cohort			After propensity score matching		
	Low-CONUT (*n* = 275)	High-CONUT (*n* = 147)	*p* value	Low-CONUT (*n* = 135)	High-CONUT (*n* = 135)	*p* value
Age (years)						
Median (range)	69 (41–96)	70 (22–86)	0.94	70 (43–85)	70 (22–86)	0.58
Sex						
Male	151	85	0.57	71	78	0.39
Female	124	62		64	57	
Smoking history						
Never	100	44	0.18	49	44	0.52
Ever	175	103		86	91	
Performance status						
0	252	125	0.04	122	121	0.84
1–3	23	22		13	14	
CONUT score						
Median (range)	1 (0–1)	2 (2–6)	< 0.01	1 (0–1)	2 (2–6)	< 0.01
Operation						
Pneumonectomy	2	3	0.23	0	3	0.16
Lobectomy	233	117		116	110	
Segmentectomy	13	5		8	5	
Wedge resection	27	22		11	17	
Histology						
Adenocarcinoma	211	102	0.15	105	96	0.21
Others	64	43		30	39	
Pathological stage						
I	209	100	0.07	98	99	0.70
II	39	34		27	23	
III	27	13		10	13	
Adjuvant chemotherapy						
None	221	115	0.67	109	106	0.90
Oral anticancer drug	28	14		12	13	
Platinum doublet	26	18		14	16	
Pleural invasion						
None	213	101	0.01	98	97	0.89
Present	62	42		37	38	
Vascular invasion						
None	179	82	< 0.01	84	79	0.53
Present	96	60		51	56	
Lymphatic invasion						
None	201	102	0.09	98	97	0.89
Present	73	41		37	38	
Pulmonary metastasis						
None	270	135	< 0.01	130	131	0.73
Present	5	8		5	4	

*CONUT* controlling nutritional status

**Table 2:** Summary of the prognoses

Prognosis	Entire cohort			After propensity score matching		
	Low-CONUT (*n* = 275)	High-CONUT (*n* = 147)	*p* value	Low-CONUT (*n* = 135)	High-CONUT (*n* = 135)	*p* value
Recurrence	60 (21.8%)	43 (29.3%)	0.23	39 ( 28.9%)	37 (27.4%)	0.19
Locoregional	25 (41.7%)	12 (27.9%)		20 (51.3%)	12 (32.4%)	
Distant	21 (35.0%)	21 (48.8%)		11 (28.2%)	17 (46.0%)	
Both	14 (23.3%)	9 (20.9%)		8 (20.5%)	7 (18.9%)	
Unknown	0 (0.0%)	1 (2.3%)		0 (0.0%)	1 (2.7%)	
Total death	51 (18.6%)	35 (23.8%)	0.38	30 (22.2%)	32 (23.7%)	0.61
Lung cancer death	28 (54.9%)	20 (57.1%)		19 (63.3%)	17 (53.1%)	
Other cancer death	6 (11.8%)	7 (20.0%)		4 (13.3%)	7 (21.9%)	
Non-malignant disease	13 (25.5%)	5 (14.3%)		6 (20.0%)	6 (18.8%)	
Unknown	4 (7.8%)	2 (5.7%)		1 (3.3%)	2 (6.3%)	

*CONUT* controlling nutritional status

### Survival analysis

**Fig. 3:**
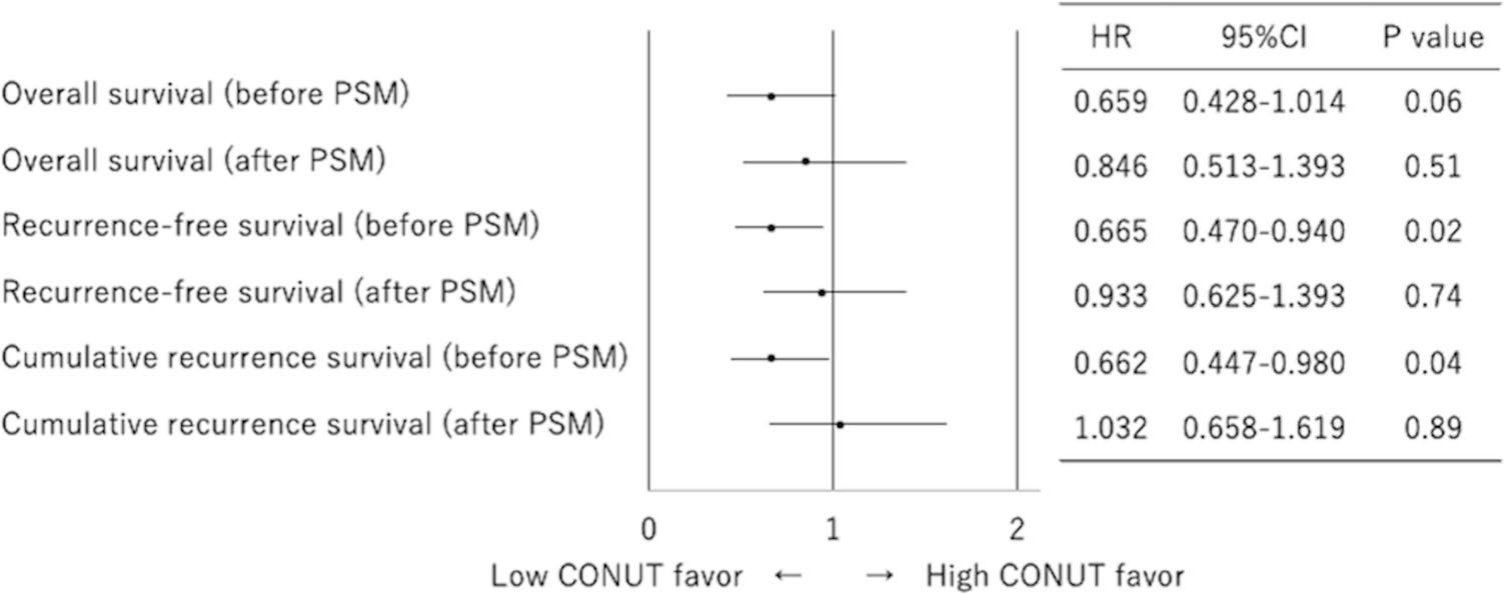
Forest plots about overall survival, recurrence-free survival, and cumulative recurrence rate at each controlling nutritional status. Before (**A**) and after (**B**) propensity score matching. *PSM* propensity score matching, *CONUT* controlling nutritional status, *HR* hazard ratio, *CI* confidence interval

The median follow-up period was 5.7 years for all patients and 6.2 years for censored patients. Patients with low CONUT scores tended to have better OS than those with high CONUT scores in the whole cohort (HR, 0.659; 95% CI 0.428–1.014; *p* = 0.04) (Fig. 3). Patients with low CONUT scores also had better RFS than those with high CONUT scores (HR, 0.665; 95% CI 0.470–0.940; *p* = 0.02). In addition, patients with low CONUT scores had a better CRR than those with high CONUT scores (HR, 0.662; 95% CI 0.447–0.980; *p* = 0.04). In the multivariate analysis for CRR, pathological stage and pleural invasion were independent prognostic factors, while CONUT score was not (Table 3).

**Table 3:** Multivariate analysis of factors associated with cumulative recurrence

Factors	HR (95% CI)	*p* value
Age (years)	1.008 (0.985–1.033)	N/A
Sex		
Male		
Female	0.860 (0.497–1.490)	0.59
Smoking history		
Never		
Ever	0.899 (0.497–1.490)	0.73
Performance status		
0		
1–3	0.999 (0.498–2.006)	1.00
CONUT score		
0–1		
≥ 2	1.291 (0.851–1.960)	0.23
Operation		
Lobectomy or more		
Limited resection	1.627 (0.878–3.017)	0.12
Histology		
Adenocarcinoma		
Others	1.295 (0.777–2.158)	0.32
Pathological stage		
I		
II–III	3.216 (1.900–5.442)	< 0.01
Pleural invasion		
None		
Present	1.599 (1.002–2.553)	0.05
Vascular invasion		
None		
Present	1.292 (0.723–2.308)	0.39
Lymphatic invasion		
None		
Present	1.643 (0.965–2.799)	0.07
Pulmonary metastasis		
None		
Present	N/A	

*CONUT* controlling nutritional status, *HR* hazard ratio, *CI* confidence interval, *N/A* not available

### Propensity score matching

After propensity score matching, there were 135 patients each in the low-CONUT and high-CONUT groups. The clinicopathological characteristics of the patients after PSM (male, *n* = 71; female, *n* = 64; median age, 70 years; range 22–86 years) are shown in Table 1. There were no significant differences in patient characteristics between the low-CONUT and high-CONUT groups.

**Fig. 4:**
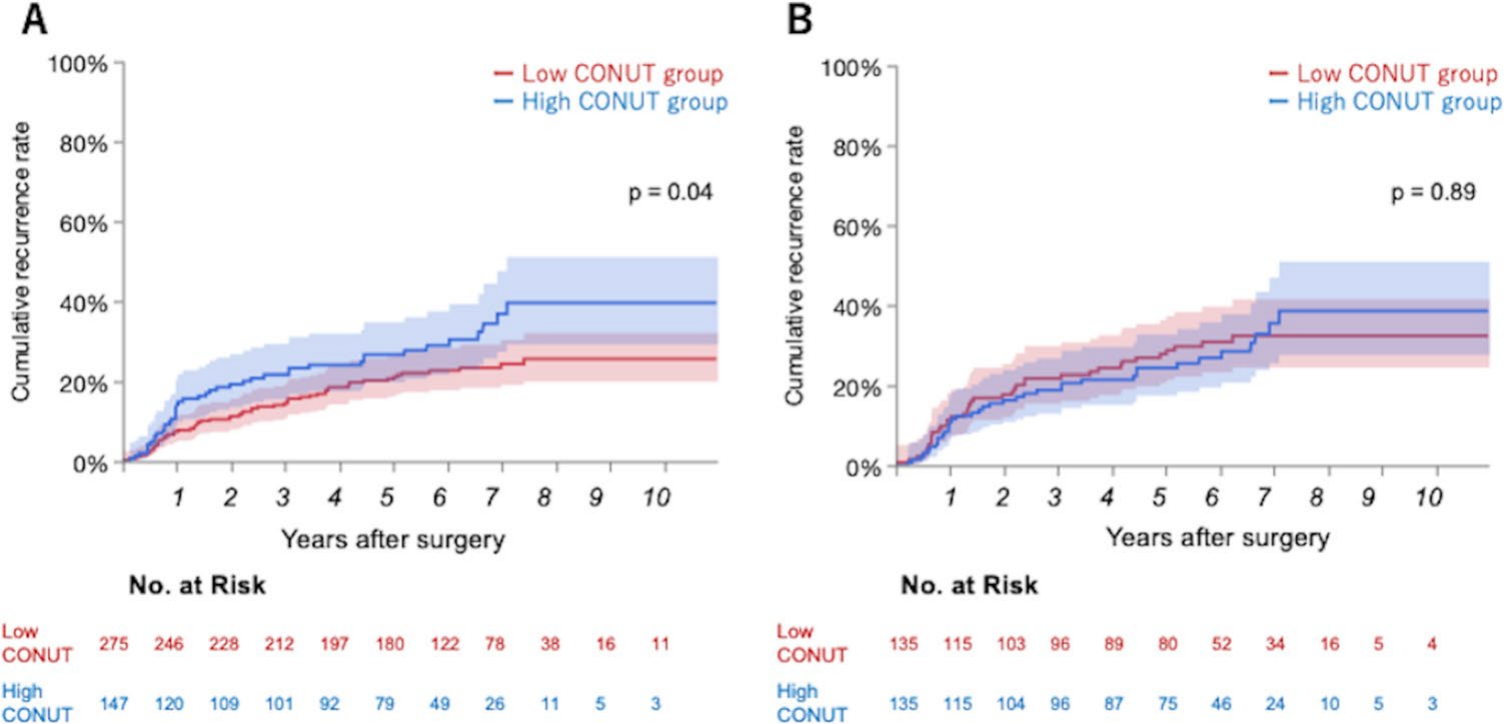
Cumulative recurrence rate before propensity score matching (**A**) and after (**B**). *CONUT* controlling nutritional status

There were no significant differences in OS, RFS, or CRR between patients with low and high CONUT scores after PSM (OS: HR 0.846, 95% CI 0.513–1.393, *p* = 0.51; RFS: HR 0.933, 95% CI 0.625–1.393, *p* = 0.74; CRR: HR 1.032, 95% CI 0.658–1.619, *p* = 0.89) (Figs. 3 and 4).

## Discussion

In recent years, the evaluation of nutritional status as a prognostic factor for cancer has attracted attention. For example, the prognostic nutritional index, a nutritional assessment, has been reported to be a prognostic factor for various solid tumors [17, 18]. Indicators such as the neutrophil-to-lymphocyte ratio have also been reported to be associated with the prognosis of lung cancer [19]. The CONUT score is a prognostic marker for several cancers [20–23]. The CONUT score was also reported to be a prognostic factor in lung cancer [13, 24]. However, most of these studies used survival as the endpoint. The nutritional status is a reflection of overall health, even in patients without cancer, and is likely to be affected by non-cancer diseases. Therefore, the use of survival as an endpoint may not assess the true cancer prognosis. In this study, we evaluated the usefulness of the CONUT score as a prognostic factor using the cumulative recurrence rate rather than survival as the endpoint.

In our study, the high-CONUT group had significantly higher rates of pleural invasion, vascular invasion, and pulmonary metastasis. This may be attributed to suppressed anticancer immunity in patients with high CONUT scores because the nutritional status has been reported to be related to the immune status [25]. Another possibility is that the high malignancy of cancer is responsible for the nutritional status of patients with high CONUT scores. Further studies are warranted to confirm these findings.

In the whole cohort, the OS, RFS, and CRR were significantly worse in patients with high CONUT scores, as previously reported. However, the pathological grade of the cancer was also high in patients with high CONUT scores, and we added a PSM analysis to eliminate bias as much as possible. As a result, the CONUT score was not an independent prognostic factor for OS, RFS, or CRR. Therefore, nutritional assessment using the CONUT score may be a predictor of the systemic status after pulmonary resection, rather than the prognosis of cancer.

The CONUT score was not identified as a risk factor for recurrence in the present study. Although several reports have examined the association between the CONUT score and OS or RFS [13–15], few have examined the association between the CONUT score and the recurrence rate in patients with lung cancer. Shoji et al. reported that in pathologic stage I NSCLC, the group with high CONUT scores had significantly shorter OS, RFS, and cancer-specific survival in comparison to the group with low CONUT scores [14]. However, their study population was limited to patients with early-stage lung cancer, and only included 138 cases. They also did not consider the differences in characteristics between the low- and high-CONUT groups. We used PSM to eliminate bias as much as possible, and we believe this is why our results differ from those of previous studies.

### Limitations

The present study was associated with several limitations. First, although PSM eliminated selection bias as much as possible, this was a retrospective study and a selection bias could not be completely eliminated. In addition, 114 patients were missing preoperative total cholesterol values, and the CONUT score could not be calculated for these patients. This may have resulted in a selection bias. Due to the limitations of this retrospective study, a prospective observational study may be necessary for an accurate evaluation. Second, this was a single-center study. To confirm the relationship between the CONUT score and the pathological grading of lung cancer, the results of this study need to be validated in multiple centers.

## Conclusions

This study suggests a relationship between the CONUT score and the pathological grading of lung cancer. Patients with a poor nutritional status, as judged by the CONUT score, may be at high risk for recurrence owing to the high frequency of pleural invasion, vascular invasion, and lung metastasis. It is necessary to establish optimal perioperative treatment strategies, including neoadjuvant chemotherapy with molecularly targeted agents and ICIs and to select appropriate surgical techniques for such patients.

## Abbreviations

NSCLC: Non-small cell lung carcinoma
OS: Overall survival
DFS: Disease free survival
PET: Positron emission tomography
ICI: Immune checkpoint inhibitor
CONUT: Controlling nutritional status
CT: Computed tomography
MRI: Magnetic resonance imaging
RFS: Recurrence-free survival
CRR: Cumulative recurrence rate
HR: Hazard ratio
CI: Confidence interval
PSM: Propensity score matching
PS: Performance status
